# Supplementary material for: Serovars, Virulence and Antimicrobial Resistance Genes of Non-Typhoidal Salmonella Strains from Dairy Systems in Mexico
Source: Antibiotics (Basel). 2023 Nov 25;12(12):1662. doi: 10.3390/antibiotics12121662 (PMC10740734; doi:10.3390/antibiotics12121662)
Supplement: Supplementary file 1 [file antibiotics-12-01662-s001.zip › antibiotics-2687815-supplementary.pdf]

Supplementary Materials

Table S1. Characteristics of the genomes and the assemblies of the strains of *Salmonella enterica* studied. GC: is the percentage of bases Guanine and Cytosine. N50 is the length of the shortest contig for which longer and equal length contigs cover at least 50 % of the assembly.

Sample ID	Number of reads	Total length	GC (%)	Coverage	Number of Contigs	Number of Contigs > 1000 bp	N50
SEDGO167B	776,737	4,593,846	52.24	84.9	40	20	423,601
SEAGS147B	526,530	4,589,491	52.24	57.6	42	25	268,766
SECOA310PC	2,457,975	4,641,077	52.08	265.9	52	43	230,122
SEDGO304PC	2,316,740	4,583,641	52.25	253.7	77	38	249,025
SEDGO294PC	1,999,364	4,623,514	52.26	217.1	58	32	683,919
SECOA322PC	660,221	4,492,678	52.29	73.8	43	23	390,322
SEDGO298PC	221,338	4,747,819	52.17	23.4	113	81	86,578
SEDGO269B	2,188,845	4,904,065	52.10	224.1	200	123	72,404
SECOA255PC	3,012,059	4,844,097	52.08	312.1	67	30	254,933
SEDGO276B	1,530,051	4,806,726	52.13	159.8	80	23	464,704
SECOA252B	366,610	4,830,844	52.04	38.1	61	32	308,114
SEDGO318B	2,410,367	4,782,667	52.23	253.0	82	37	399,826
SECOA320C	444,056	4,767,606	52.22	46.8	104	46	225,681
SECOA225PC	1,184,561	4,622,798	52.21	128.6	47	16	695,405
SECOA246B	1,225,533	4,623,994	52.21	133.0	49	14	533,824
SECOA243PC	4,625,764	4,633,902	52.22	501.1	69	20	473,762
SESLP86C	3,315,383	4,843,271	52.15	343.6	70	27	728,136
SETLX55C	3,471,853	4,666,274	52.22	373.5	47	33	288,006
SECOA245B	1,678,319	4,757,859	52.26	177.1	83	49	240,267
SEBC65B	3,637,120	4,669,748	52.22	391.0	70	28	478,611
